# Postoperative recurrence prediction model for perianal abscess using machine learning algorithms

**DOI:** 10.3389/fpubh.2025.1722109

**Published:** 2025-12-04

**Authors:** Dawei Wang, Caixia Zhang, Zhiran Li, Zheng Zheng, Ao Chen, Yuan Fang, Shaohua Huangfu, Chungen Zhou, Qizhi Liu, Bin Jiang

**Affiliations:** 1 National Colorectal Disease Center, Nanjing Hospital of Chinese Medicine, Affiliated to Nanjing University of Chinese Medicine, Nanjing, Jiangsu, China; 2 Guang’anmen Hospital, China Academy of Chinese Medical Sciences, Beijing, China; 3 Xuzhou City Hospital of TCM, Xuzhou, China; 4 Nanjing Hospital of Chinese Medicine Affiliated with Nanjing University of Chinese Medicine, Nanjing, China

**Keywords:** perianal abscess, recurrence, machine learning, CatBoost, Shap

## Abstract

**Objective:**

This study aimed to develop a machine learning–based model to predict recurrence risk after perianal abscess surgery, thereby supporting personalized follow-up and intervention strategies.

**Methods:**

Clinical data were collected from patients with perianal abscess who underwent surgery at Nanjing Hospital of Chinese Medicine, Affiliated to Nanjing University of Chinese Medicine between January 2022 and June 2023. Significant predictors were identified using the least absolute shrinkage and selection operator (LASSO) algorithm combined with multivariate logistic regression. The Synthetic Minority Over-sampling Technique (SMOTE) was applied to balance class distribution, and several machine learning (ML) algorithms were employed for model construction. Model performance was evaluated by the area under the receiver operating characteristic curve (AUC), sensitivity, specificity, and accuracy. Model calibration was assessed using calibration curves. The effectiveness was evaluated through Decision Curve Analysis (DCA). Finally, the SHapley Additive ExPlanations (SHAP) were used to interpret the best-performing model and quantify the contribution of each predictor to its predictions.

**Results:**

A total of 737 patients with perianal abscess were included in the study. A history of diabetes, abscess space, and the aggregate index of systemic inflammation (AISI) were identified as the three strongest predictors of recurrence. Among all evaluated models, the CatBoost model showed the highest discriminatory power in the training set (AUC = 0.821, 95% *CI*: 0.777–0.864), validation set (AUC = 0.744, 95% *CI*: 0.616–0.872), and temporal validation set (AUC = 0.735, 95% *CI*: 0.649–0.821).

**Conclusion:**

The machine learning–based model effectively identifies patients at high risk of recurrence after perianal abscess surgery. The CatBoost model achieved the best predictive performance, while SHAP analysis enhanced interpretability, supporting individualized patient management.

## 1 Introduction

A perianal abscess is an acute infection of the anal glands caused by ductal obstruction and may extend into subcutaneous, intersphincteric, ischiorectal, or supralevator spaces (1). It typically presents with sudden onset and severe pain, and surgery remains the mainstay of treatment (2–4). The primary treatment objective is to ensure effective drainage and preserve sphincter integrity, thereby minimizing recurrence (5, 6). However, recurrence rates remain high—approximately 30% according to some studies (7)—which markedly affects patients’ long-term quality of life. Therefore, identifying high-risk patients early and accurately is crucial for tailoring surgical strategies and optimizing postoperative management.

Previous research has reported associations between recurrence and factors such as abscess extension, operative technique, and diabetes history (8–13). However, most previous studies have relied on traditional statistical methods such as logistic regression, which struggle to capture nonlinear relationships and variable interactions in complex clinical data. With the advancement of artificial intelligence, ML algorithms have shown remarkable advantages in predicting medical outcomes (14). Unlike traditional methods, ML techniques—such as random forests and gradient boosting—can autonomously learn complex patterns, enabling more accurate risk stratification. Despite their strong predictive ability, ML models are often criticized for their “black-box” nature, which limits clinical interpretability and trust. SHAP, a game theory–based approach, quantifies the contribution of each feature to model predictions, thereby enhancing transparency and interpretability.

Although ML-based models have been applied to recurrence prediction in surgical and infectious disease contexts (15, 16), no study has yet integrated ML with the SHAP framework to predict postoperative recurrence of perianal abscess. This study aims to bridge this gap by incorporating multiple clinical indicators from the perioperative period and introducing AISI as a novel inflammatory biomarker. A range of ML algorithms were applied to develop a predictive model for postoperative recurrence, and SHAP was used to visualize and explain the contribution of key features in the optimal model. By developing an interpretable ML model, we seek to provide clinicians with a practical decision-support tool to facilitate personalized and precision management of perianal abscess.

## 2 Materials and methods

### 2.1 Data source

A total of 737 patients diagnosed with perianal abscess and meeting the inclusion and exclusion criteria were enrolled at Nanjing Hospital of Traditional Chinese Medicine between January 2022 and June 2023. All surgical procedures were performed by experienced colorectal surgeons. All operations were conducted according to standardized protocols to ensure optimal postoperative recovery and minimize recurrence risk. Model development and validation followed the TRIPOD (Transparent Reporting of a multivariable prediction model for Individual Prognosis Or Diagnosis) guidelines. Clinical data were extracted from the electronic medical records system. The study was approved by the Research Ethics Committee of Nanjing Hospital of Chinese Medicine, Nanjing University of Chinese Medicine (approval No. KYS2024060) and conducted in accordance with the Declaration of Helsinki. As anonymized retrospective data were used, the requirement for informed consent was waived by the Research Ethics Committee of Nanjing Hospital of Chinese Medicine, affiliated with Nanjing University of Chinese Medicine.

### 2.2 Inclusion and exclusion criteria

#### 2.2.1 Inclusion criteria

Patients diagnosed with perianal abscess according to established diagnostic criteria (17), aged ≥18 years, who underwent incision and drainage or seton placement, and had complete clinical data available.

#### 2.2.2 Exclusion criteria

Patients with severe comorbidities (e.g., malignancy, significant cardiopulmonary dysfunction), immunocompromised status (including long-term immunosuppressive therapy), inflammatory bowel disease (Crohn’s disease or ulcerative colitis), recent anorectal or colorectal surgery, perianal skin or sebaceous gland infections, coagulopathy, severe infections in other organs, or pregnancy/lactation.

### 2.3 Study variables and outcome definitions

Based on clinical relevance and prior studies, the collected variables included sex, age, history of diabetes, body mass index (BMI), history of abscess, abscess space, and surgical approach. Surgical procedures were classified into two modalities: incision and drainage, and incision with seton placement. Abscess classification was determined using preoperative imaging—three-dimensional (3D) anorectal ultrasonography or pelvic/anal MRI—and intraoperative exploration, referencing the puborectal plane. Abscesses located above this plane were classified as high-position, whereas those below it were classified as low-position (18). Additionally, serum biomarkers such as total cholesterol (TC), aggregate index of systemic inflammation (AISI) [neutrophil × monocyte × platelet/lymphocyte], lymphocyte-to-high-density lipoprotein ratio (LHR) [lymphocyte/high-density lipoprotein], neutrophil-to-lymphocyte ratio (NLR) [neutrophil/lymphocyte], systemic inflammatory response index (SIRI) [neutrophil × monocyte/lymphocyte], and systemic immune-inflammation index (SII) [platelet × neutrophil/lymphocyte] were measured. All serum biomarkers were analyzed using venous blood samples obtained within 24 h prior to surgery. All analyses were performed by the hospital’s laboratory department in strict accordance with standard protocols to ensure methodological consistency. Patients were followed up for 1 year after surgery. Recurrence was defined as the reappearance of perianal abscess symptoms—such as redness, pain, swelling, ulceration, or purulent discharge—within 1 year, confirmed by color Doppler 3D ultrasound.

### 2.4 Data preprocessing and variable selection

All variables were complete, with no missing data observed. Outliers detected in continuous variables (e.g., AISI) were not removed, considering their clinical importance and the robustness of tree-based algorithms to such variations. Continuous variables were normalized via *Z*-score transformation to ensure comparability across different scales. Categorical variables were encoded as numerical values using label encoding (e.g., “no/female/low” = 0; “yes/male/high” = 1).

The initial dataset contained 13 features: 5 categorical and 8 continuous variables. To reduce overfitting and maintain clinical relevance, feature selection was first performed using LASSO regression. The optimal regularization parameter (*λ*) was determined through 10-fold cross-validation, and the most predictive variables, identified by non-zero coefficients, were selected based on the coefficient trajectory and the cross-validation error curve. Subsequently, multicollinearity among the LASSO-selected variables was assessed using the Variance Inflation Factor (VIF). The VIF results (<5) indicated the absence of multicollinearity, supporting the robustness of subsequent statistical modeling. Multivariate logistic regression was applied to explore group differences, and predictors reaching statistical significance (*p* < 0.050) were incorporated into the final model.

### 2.5 Modeling method

#### 2.5.1 Data splitting and sample size estimation

The dataset was chronologically divided into two time periods: January 2022–December 2022 and January 2023–June 2023. Data from January 2022–December 2022 were further divided into a training set and an internal validation set at a 7:3 ratio, while data from January 2023–June 2023 were used as the temporal validation set. The training set was used to develop machine learning models, whereas the internal validation set was employed to evaluate and compare model performance. The temporal validation set was used to assess model robustness over time, accounting for potential changes in clinical practice or patient characteristics. Sample adequacy was ensured using the classical “events per variable” criterion (19, 20), which indicated a minimum total sample size of 207 cases. In total, 737 patients were included, of whom 531 were assigned to the training and internal validation sets, exceeding the minimum requirement and ensuring model robustness. Additionally, we used the sample size calculation approach for prediction models proposed by Riley et al. (21), which yielded a minimum requirement of 538 participants. With 531 participants included, our study nearly met this standard, supporting the stability and generalizability of the model.

#### 2.5.2 Model construction, optimization, and evaluation

The selected predictors were used to construct nine machine learning models, including logistic regression, support vector machine (SVM), *k*-nearest neighbors (KNN), gradient boosting machine (GBM), AdaBoost, neural networks, XGBoost, CatBoost, and LightGBM. Model hyperparameters were optimized via repeated 10-fold cross-validation (five iterations), aiming to maximize the average AUC as the evaluation metric. The SMOTE (with parameter settings: *k*=5, perc.over=100, perc.under=200) was applied to balance the dataset and mitigate class imbalances. The grid search parameter ranges for each algorithm are summarized in Supplementary Table S1. Performance metrics included the AUC, sensitivity, specificity, and accuracy. Calibration Curves and DCA were jointly employed for model evaluation. Calibration Curves assessed the calibration accuracy by visualizing the fit of predicted probabilities to actuals. Meanwhile, DCA was further performed to evaluate the clinical utility of the models. To interpret the best-performing “black-box” model, SHAP analysis was applied. Feature importance was quantified using the mean absolute SHAP values, and SHAP summary plots were generated to visualize the contribution and distribution of key features across the dataset.

### 2.6 Statistical analysis

All data analyses and graphical visualizations for model construction were conducted using R software (version 4.3.2). Quantitative data with normal distribution were presented as mean ± standard deviation (x ± s), and differences between groups were assessed using the independent-samples *t*-test. Non-normally distributed data were expressed as median (*P25, P75*) and compared using the Mann–Whitney *U* test. Categorical variables were expressed as frequencies and percentages [*n* (%)] and compared using the chi-squared (*χ^2^*) test. Statistical significance was defined as a *p*-value less than 0.05.

## 3 Results

### 3.1 Baseline characteristics

**Figure 1:**
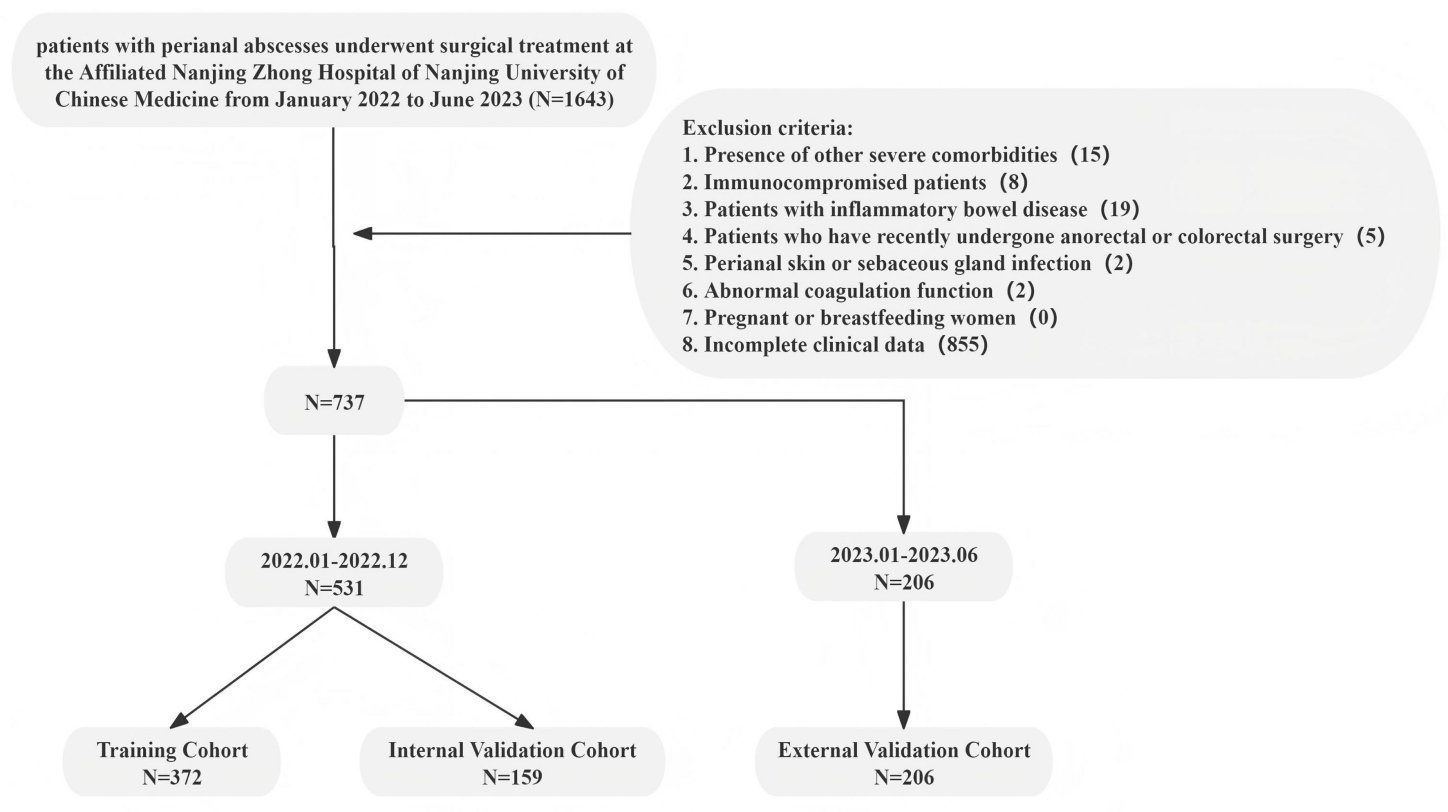
Flow chart of a prediction model for perianal abscess recurrence.

This study initially screened 1,643 patients diagnosed with perianal abscesses. Following the application of the exclusion criteria outlined in Figure 1, 737 patients were ultimately included in the analysis. Of these, 531 patients (January–December 2022) were allocated to the training and internal validation sets, and 206 patients (January–June 2023) constituted the temporal validation set. Table 1 summarizes the baseline characteristics of patients in the internal set and the temporal validation set. Among all baseline variables, only LHR and surgical approach demonstrated statistically significant differences (*p* < 0.050). In general, the internal set and the temporal validation set were largely comparable, despite some variations in baseline characteristics.

**Table 1:** Baseline characteristics of the internal set and the temporal validation set.

Variables	Total (*n* = 737)	The internal set (*n* = 531)	The temporal validation set (*n* = 206)	*p*-value
Age, Mean ± SD	37.07 ± 11.61	36.85 ± 11.49	37.62 ± 11.91	0.422
BMI, Mean ± SD	25.96 ± 4.54	26.02 ± 4.49	25.81 ± 4.67	0.585
TC, Mean ± SD	4.86 ± 0.99	4.84 ± 0.99	4.93 ± 0.99	0.225
LHR, Mean ± SD	1.45 ± 0.63	1.48 ± 0.65	1.36 ± 0.55	0.022
NLR, Mean ± SD	5.58 ± 3.93	5.53 ± 3.77	5.69 ± 4.33	0.624
SIRI, Mean ± SD	4.25 ± 4.20	4.23 ± 4.15	4.31 ± 4.31	0.806
SII, Mean ± SD	1413.65 ± 984.24	1391.57 ± 963.88	1470.55 ± 1035.13	0.329
AISI, M (Q_1_, Q_3_)	766.54 (348.76, 1398.40)	753.93 (361.03, 1375.70)	803.16 (318.82, 1473.84)	0.665
Gender, *n* (%)				0.469
Female	104 (14.11)	78 (14.69)	26 (12.62)	
Male	633 (85.89)	453 (85.31)	180 (87.38)	
Diabetes, *n* (%)				0.874
No	667 (90.50)	480 (90.40)	187 (90.78)	
Yes	70 (9.50)	51 (9.60)	19 (9.22)	
History of Perianal Abscess, *n* (%)				0.819
No	688 (93.35)	495 (93.22)	193 (93.69)	
Yes	49 (6.65)	36 (6.78)	13 (6.31)	
Abscess space, *n* (%)				0.406
Low position	692 (93.89)	501 (94.35)	191 (92.72)	
High position	45 (6.11)	30 (5.65)	15 (7.28)	
Surgical modalities, *n* (%)				0.011
Abscess incision and drainage	660 (89.55)	466 (87.76)	194 (94.17)	
Abscess incision and seton placement	77 (10.45)	65 (12.24)	12 (5.83)	

The baseline clinical characteristics of the two groups are presented in Table 2. At the one-year follow-up, perianal abscess recurrence occurred in 20.49% of patients (151/737). There were no significant inter-group differences in gender, age, or BMI (*p* > 0.050). Male patients comprised 85.89% of those who experienced recurrence.

**Table 2:** Baseline clinical characteristics of the different patient groups.

Variables	Total (*n* = 737)	Non-recurrence group (*n* = 586)	Recurrence group (*n* = 151)	*p*-value
Age, Mean ± SD	37.07 ± 11.61	37.00 ± 11.44	37.32 ± 12.26	0.764
BMI, Mean ± SD	25.96 ± 4.54	25.96 ± 4.64	25.96 ± 4.16	1.000
TC, Mean ± SD	4.86 ± 0.99	4.84 ± 0.88	4.95 ± 1.32	0.343
LHR, Mean ± SD	1.45 ± 0.63	1.45 ± 0.61	1.42 ± 0.67	0.553
NLR, Mean ± SD	5.58 ± 3.93	5.30 ± 3.33	6.64 ± 5.59	0.005
SIRI, Mean ± SD	4.25 ± 4.20	3.92 ± 3.70	5.50 ± 5.58	0.001
SII, Mean ± SD	1413.65 ± 984.24	1348.80 ± 907.94	1665.31 ± 1207.49	0.003
AISI, M (Q_1_, Q_3_)	766.54 (348.76, 1398.40)	716.68 (336.71, 1260.43)	950.31 (480.73, 1834.65)	<0.001
Gender, *n* (%)				0.480
Female	104 (14.11)	80 (13.65)	24 (15.89)	
Male	633 (85.89)	506 (86.35)	127 (84.11)	
Diabetes, *n* (%)				<0.001
No	667 (90.50)	545 (93.00)	122 (80.79)	
Yes	70 (9.50)	41 (7.00)	29 (19.21)	
History of Perianal Abscess, *n* (%)				0.278
No	688 (93.35)	550 (93.86)	138 (91.39)	
Yes	49 (6.65)	36 (6.14)	13 (8.61)	
Abscess space, *n* (%)				<0.001
Low position	692 (93.89)	570 (97.27)	122 (80.79)	
High position	45 (6.11)	16 (2.73)	29 (19.21)	
Surgical modalities, *n* (%)				0.336
Abscess incision and drainage	660 (89.55)	528 (90.10)	132 (87.42)	
Abscess incision and seton placement	77 (10.45)	58 (9.90)	19 (12.58)	

### 3.2 Variable selection

**Figure 2:**
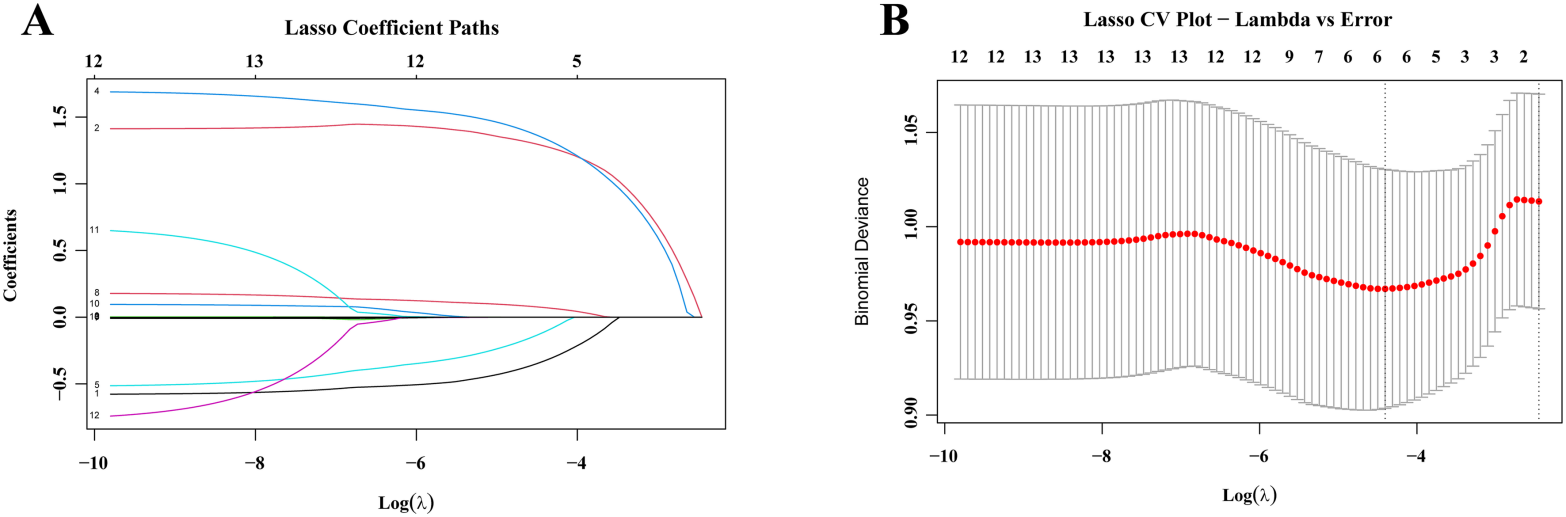
Coefficient plots and cross-validation plots for LASSO regression analyses of variables. **(A)** Number of non-zero coefficients in the model. **(B)** Number of variables corresponding to different *λ* values. Six variables were selected by LASSO regression, and constituted the basic factors of the prediction mode.

Given the relatively limited sample size, correlations between predictors may introduce multicollinearity and thus influence model stability. To address this issue, the LASSO regression algorithm was employed for variable selection, followed by 10-fold cross-validation to identify the optimal regularization parameter (*λ*). Figures 2A,B illustrate the LASSO regression coefficient path and the cross-validation curve, which identified six variables with potential predictive value: gender, diabetes history, abscess space, surgical approach, TC, and AISI. The VIF values for the six selected predictors were all <5, confirming the lack of substantial multicollinearity among them (Supplementary Table S2). Subsequent multivariate logistic regression identified abscess space, diabetes history, and AISI as independent predictors of postoperative recurrence (Table 3). Hence, the final model was constructed using only these three significant predictors, excluding those without statistical significance.

**Table 3:** Predicting Indicators Screened from LASSO Regression.

Variables	Estimate	OR	95% *CI*		*p*-value	OR	95% *CI*		*p*-value
			Lower	Upper			Lower	Upper	
Intercept	−1.9285								
Gender = Male	−0.3043	0.62	0.32	1.20	0.157				
Diabetes = Positive	1.2667	3.95	1.99	7.83	<0.001	4.71	2.33	9.51	<0.001
Abscess space = High position	1.3136	4.93	1.83	13.25	0.002	5.02	1.71	14.74	0.003
Surgical modalities = seton placement	−0.0381	0.65	0.26	1.61	0.353				
TC	0.0662	1.23	0.96	1.57	0.098				
AISI	−0.0002	1.00	1.00	1.00	0.005	1.00	1.00	1.01	0.032

### 3.3 Performance of different models

To correct the imbalance between recurrence (*n* = 75) and non-recurrence (*n* = 297) cases, the SMOTE was applied only to the training set, yielding a balanced dataset of 150 cases per class. The internal and temporal validation sets were kept unchanged to maintain realistic performance evaluation.

**Figure 3:**
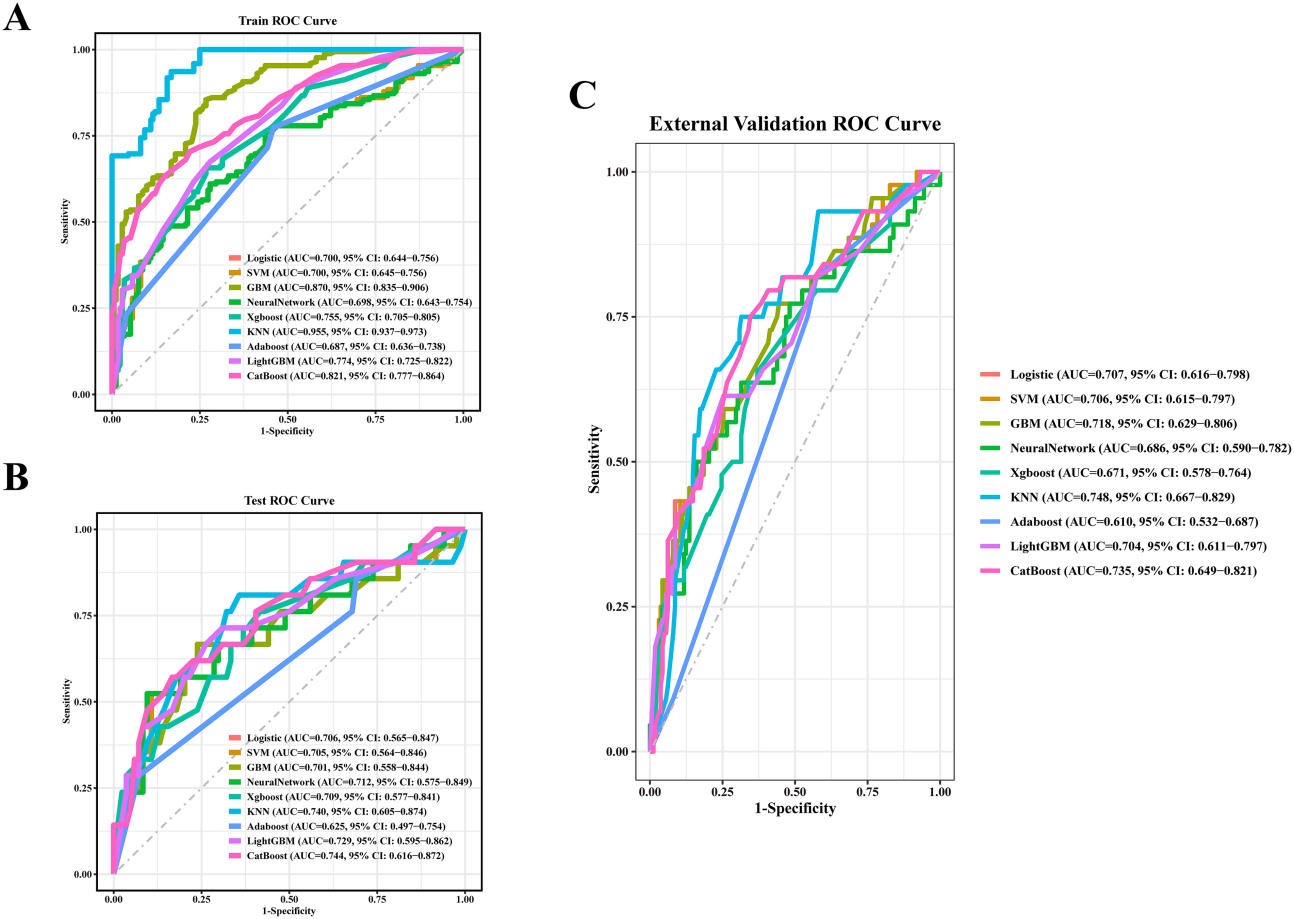
The receiver operating characteristic (ROC) curves for the models. **(A)** ROC curves for the models in the training set. **(B)** ROC curves for the models in the internal validation set. **(C)** ROC curves for the models in the temporal validation set.

**Figure 4:**
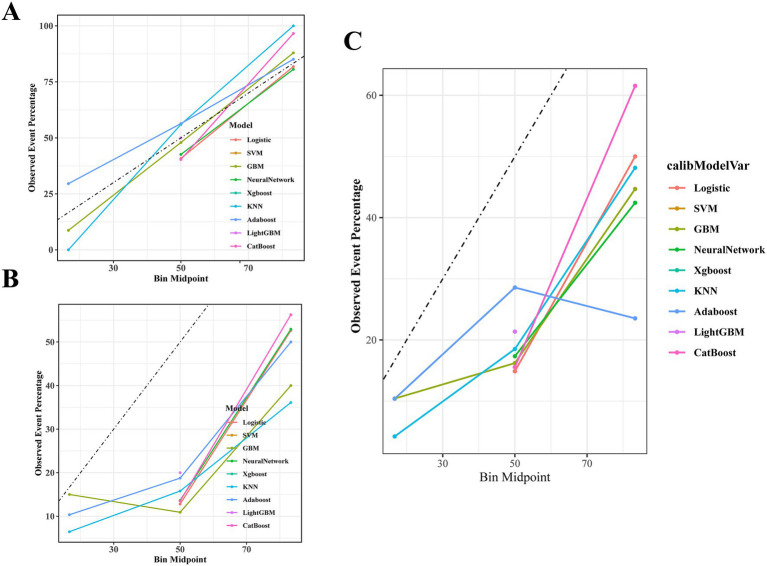
The calibration curves for the models. **(A)** The calibration curves for the models in the training set. **(B)** The calibration curves for the models in the internal validation set. **(C)** The calibration curves for the models in the temporal validation set.

**Figure 5:**
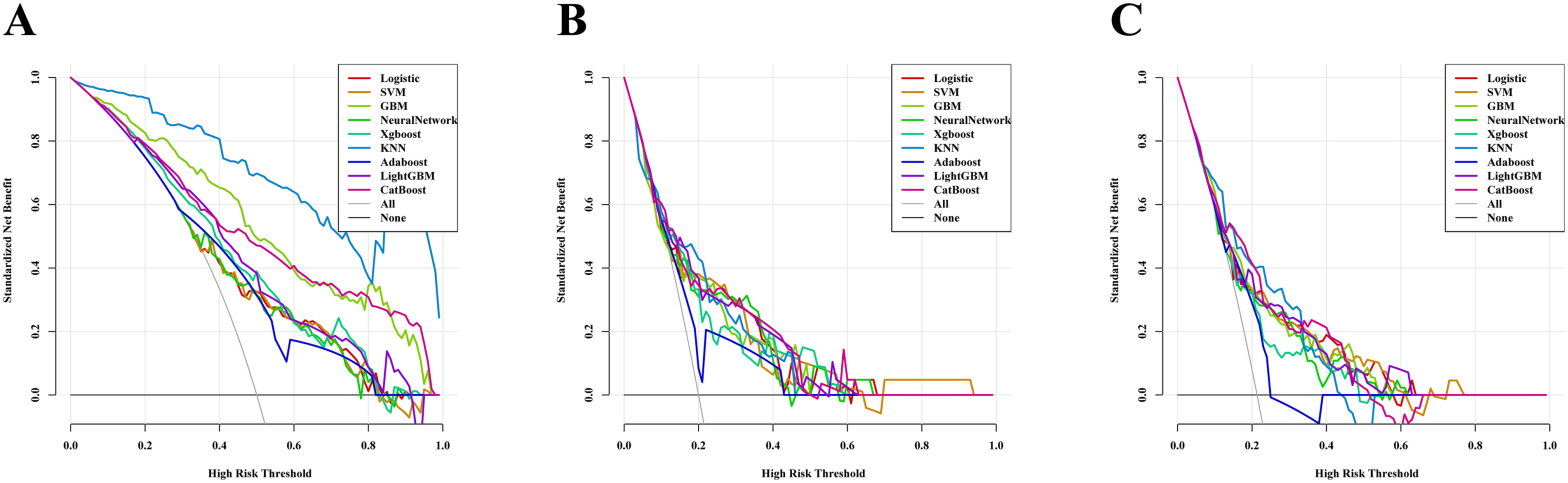
DCA curves for the models. **(A)** DCA curves for the models in the training set. **(B)** DCA curves for the models in the internal validation set. **(C)** DCA curves for the models in the temporal validation set.

The AUC values of the nine machine learning models are presented in Figure 3. Among the models, CatBoost achieved the highest predictive performance, with AUCs of 0.821 (95% *CI*: 0.777–0.864) in the training set, 0.744 (95% *CI*: 0.616–0.872) in the internal validation set, and 0.735 (95% *CI*, 0.649–0.821) in the temporal validation set. As illustrated in Figure 4, the CatBoost model most closely aligns with the ideal diagonal in the training set, internal validation set, and temporal validation set, reflecting its optimal calibration accuracy and thereby enhancing the reliability of the prediction outcomes. The DCA further reveals (Figure 5) that the CatBoost model delivers the highest standardized net benefit across a broad range of threshold probabilities, suggesting that it offers superior clinical utility compared to other models. Table 4 summarizes the accuracy, sensitivity, and specificity for each model, as derived from the confusion matrix.

**Table 4:** Metrics of the 9 machine learning models used for prediction.

Model	The training set			The internal validation set			The temporal validation set		
	Accuracy	Sensitivity	Specificity	Accuracy	Sensitivity	Specificity	Accuracy	Sensitivity	Specificity
Logistic	0.666	0.61	0.721	0.819	0.524	0.893	0.811	0.432	0.914
SVM	0.334	0.39	0.279	0.181	0.476	0.107	0.233	0.5	0.16
GBM	0.794	0.855	0.733	0.743	0.667	0.762	0.714	0.591	0.747
Neural Networks	0.666	0.61	0.721	0.829	0.524	0.905	0.767	0.5	0.84
Xgboost	0.695	0.657	0.733	0.619	0.762	0.583	0.655	0.636	0.66
KNN	0.884	0.936	0.831	0.676	0.81	0.643	0.699	0.75	0.685
Adaboost	0.657	0.773	0.541	0.8	0.286	0.929	0.51	0.818	0.426
LightGBM	0.698	0.674	0.721	0.695	0.714	0.69	0.718	0.614	0.747
CatBoost	0.741	0.703	0.779	0.781	0.571	0.833	0.675	0.75	0.654

### 3.4 Interpretability of the best model

**Figure 6:**
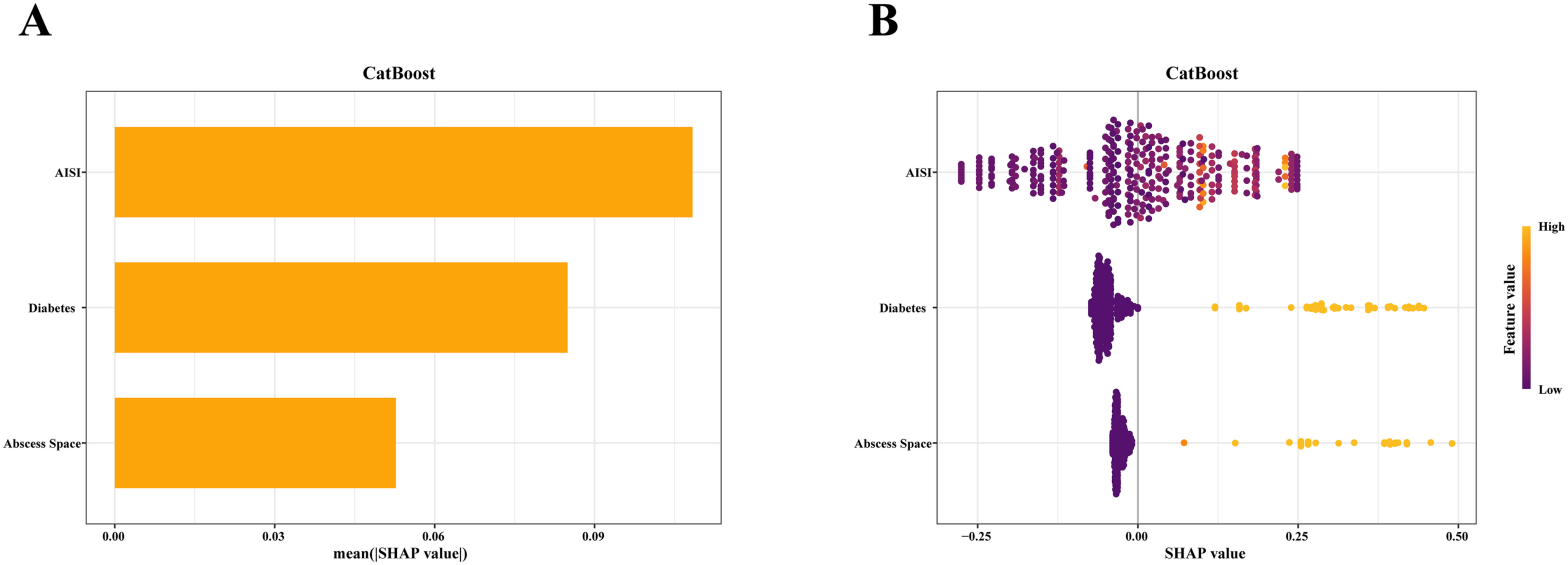
SHAP explanation plots for the CatBoost model. **(A)** SHAP Bar Plot. This chart presents the features ranked by their average SHAP absolute values, illustrating the relative importance of each variable within the model. **(B)** SHAP Bee Swarm Plot. This plot demonstrates the direction and magnitude of key features (such as AISI, diabetes, etc.) in contributing to the model output. The color gradient indicates the continuous variation of feature values, ranging from low (purple) to high (yellow).

To elucidate the internal decision process of the model, SHAP analysis was employed to quantify the relative impact of each feature on its predictive outputs. SHAP values reflect the direction and magnitude of each feature’s effect on model output, with positive values corresponding to a higher recurrence risk and negative values to a lower risk. According to Figure 6, Supplementary Table S3, feature importance in the CatBoost model ranked AISI, diabetes history, and abscess space the top three predictors, with mean absolute SHAP values of 0.1084, 0.0849, and 0.0527, respectively. The SHAP beeswarm plot offers an intuitive depiction of feature-level contributions, illustrating how different variables drive individual predictions. AISI displayed both positive and negative contributions (SHAP range: −0.276 to 0.249; 49.42% positive cases), implying a nonlinear, threshold-like effect on recurrence risk. Diabetes history exhibited mainly negative SHAP contributions (13.66% positive), reinforcing its role as a stable risk factor. Despite its relatively limited average contribution, the abscess space markedly affected specific cases, reaching a SHAP value as high as 0.490.

## 4 Discussion

This study successfully developed and validated a machine learning-based predictive model for the postoperative recurrence of perianal abscess. The results indicate that the CatBoost model, which integrates critical predictive factors such as diabetes, abscess space, and AISI, demonstrates robust predictive performance (AUC = 0.744 for the internal validation set and AUC = 0.735 for the temporal validation set).

Neutrophils, monocytes, platelets, lymphocytes, a history of diabetes, and abscess space are key factors for monitoring the prognosis and recurrence of perianal abscesses. Although individual peripheral blood immune or inflammatory markers have shown some predictive value for perianal abscess recurrence, their predictive power remains limited (22–24). A previous study that incorporated AISI into a model for predicting postoperative infection after posterior lumbar fusion surgery demonstrated its potential relevance in infectious diseases (25). In the present study, by integrating AISI into the machine learning framework, we identified a nonlinear association between AISI and the occurrence, progression, and recurrence of perianal abscess.

Diabetes remains a clinically important factor influencing both the healing process and long-term recurrence of perianal abscess (26–30). Our findings further indicate that even patients with prediabetes or suboptimal glycemic control face a markedly increased risk of recurrence. As recurrence is closely linked to the anatomical location and depth of the abscess, the abscess space serves as a distinctive parameter for perianal abscess evaluation. Incorporating this variable into the predictive model enables a more accurate estimation of postoperative recurrence risk. Multivariate logistic regression analysis revealed that abscess space was significantly associated with perianal abscess recurrence, likely due to a larger infected area and more complex infectious foci (31). Therefore, meticulous preoperative evaluation of abscess space, along with precise intraoperative management of internal fistulous tracts, may substantially improve postoperative outcomes in perianal abscess patients.

Traditional statistical models primarily identify linear associations, whereas ensemble algorithms such as CatBoost used in this study are better suited to capturing complex nonlinear relationships and interactions among variables. Compared with the model developed by Constantine et al. for predicting postoperative complications following laparoscopic colectomy (32) and the surgical site infection risk model proposed by Liu et al. (33) for lumbar surgery, the present study not only enhanced methodological rigor through temporal validation but, more importantly, integrated the SHAP framework and incorporated the novel biomarker AISI, thereby improving both model performance and clinical relevance while enhancing interpretability.

Despite these strengths, several limitations should be noted. First, this was a single-center retrospective study, and although temporal validation was employed to assess model stability, it may not fully reflect performance across different clinical settings (34, 35). Second, the limited sample size, despite using the LASSO algorithm to mitigate overfitting, limits statistical power and may affect the model’s external validity and reliability (36, 37). Third, while restricting the SMOTE algorithm to the training set helped reduce overfitting, its reliance on synthetic interpolation may still limit generalizability to real-world data (16). Fourth, institutional treatment protocols could have influenced the results, despite standardized surgical procedures and postoperative care. Therefore, external validation across multiple centers is warranted. Lastly, the clinical application of machine learning models involves ethical considerations such as fairness, transparency, and accountability, which should be further explored in future studies (38, 39).

Notably, by employing the SHAP explanation framework, we were able to transform the model’s “black-box” nature into clinically interpretable insights, highlighting its potential to enhance transparency and support clinical decision-making. Furthermore, this predictive model can be seamlessly integrated into hospital electronic medical record systems, facilitating the automatic extraction of key indicators and the real-time calculation of risk scores. This integration enables an intelligent preoperative assessment of recurrence risk and provides clinicians with valuable support in crafting personalized surgical plans and follow-up strategies, thereby advancing the individualized, precise management of perianal abscesses.

## 5 Conclusion

This study is the first to integrate machine learning with the SHAP interpretability framework for predicting the recurrence of perianal abscesses, resulting in a high-performance and explainable model. By addressing the “black box” limitation, the model provides clinically meaningful insights into recurrence risk. These findings highlight its potential as a decision-support tool for personalized management of perianal abscesses, although further multicenter validation is warranted.

## Data Availability

The original contributions presented in the study are included in the article/Supplementary material, further inquiries can be directed to the corresponding author.

## Glossary

AISI: aggregate index of systemic inflammation
BMI: body mass index
GBM: gradient boosting machine
KNN: *k*-nearest neighbors
LR: logistic regression
LASSO: least absolute shrinkage and selection operator
ML: machine learning
NLR: neutrophil-to-lymphocyte ratio
RF: random forest
SHAP: SHapley Additive ExPlanations
SVM: support vector machine
SII: systemic immune-inflammatory index
SIRI: systemic inflammatory response index
AUC: area under the receiver operating characteristic curve
LHR: lymphocyte-to-high-density lipoprotein ratio
TC: total cholesterol
TRIPOD: Transparent Reporting of a multivariable prediction model for Individual Prognosis Or Diagnosis
SMOTE: Synthetic Minority Over-sampling Technique
DCA: Decision Curve Analysis
VIF: Variance Inflation Factor
ROC: receiver operating characteristic
