# Supplementary material for: Postoperative recurrence prediction model for perianal abscess using machine learning algorithms
Source: Front Public Health. 2025 Dec 4;13:1722109. doi: 10.3389/fpubh.2025.1722109 (PMC12700084; doi:10.3389/fpubh.2025.1722109)
Supplement: Supplementary file 1 [file Supplementary_file_1.docx]

**Supplementary Table S1.Grid search ranges for each algorithm.**

| Algorithm | Key Hyperparameters | Final Configuration Value | Tuning Method |
| --- | --- | --- | --- |
| Logistic Regression | Regularization | Default parameters | No grid search was performed |
| SVM (Radial) | Sigma Cost (C) | 0.001 0.09 | Fixed parameter grid |
| GBM | Number of Trees  Tree Depth / Max Depth  Learning Rate  Minimum Samples per Leaf | 100 3 0.1 5 | Fixed parameter grid |
| Neural Networks | Hidden Layer Size  Weight Decay | 6 0.6 | Fixed parameter grid |
| XGBoost | Iterations  Max Depth  Learning Rate  Gamma  Column Sampling Rate  Min Child Weight  Row Sampling Rate | 10 3 0.001 0.5 0.5 1 0.6 | Fixed parameter grid |
| KNN | Number of Neighbors  Distance Metric  Kernel Function | 12 1 "optimal" | Fixed parameter grid |
| AdaBoost | Iterations  Max Depth  Learning Algorithm | 2 2 "Zhu" | Fixed parameter grid |
| LightGBM | Learning Rate  Number of Leaves  Max Depth  Minimum Samples per Leaf  Feature Sampling Rate  Sample Sampling Rate  L1 Regularization  L2 Regularization  Iterations | 0.05 31 6 20 0.8 0.8 0.1 0.1 5 | Manual tuning |
| CatBoost | Iterations  Use Best Model  Evaluation Metric  Ignored Features  Number of Boundaries  Tree Depth  Learning Rate | 1000 TRUE "AUC" c(4, 9) 32 6 0.05 123 | Manual tuning |

**Supplementary Table S2. Multicollinearity Test.**

| Variables | VIF |
| --- | --- |
| Gender | 1.027 |
| Diabates | 1.058 |
| Abscess Space | 1.030 |
| Surgical Modalities | 1.018 |
| TC | 1.058 |
| AISI | 1.008 |

**Supplementary Table S3. SHAP Summary Table.**

| Feature | Mean SHAP Value | Mean \|SHAP\| Value | Standard Deviation | SHAP Value Range | Proportion of Positive Impact | Importance Rank |
| --- | --- | --- | --- | --- | --- | --- |
| AISI | 0.00022 | 0.1084 | 0.1350 | -0.2756—0.2485 | 49.42% | 1 |
| Diabetes | -0.00027 | 0.0849 | 0.1285 | -0.072—0.4463 | 13.66% | 2 |
| Abscess Space | 0.00006 | 0.0527 | 0.1006 | -0.0395—0.4901 | 8.14% | 3 |
